# Unobtrusive Photoplethysmographic Monitoring Under the Foot Sole while in a Standing Posture

**DOI:** 10.3390/s18103239

**Published:** 2018-09-26

**Authors:** Seunghyeok Hong, Kwang Suk Park

**Affiliations:** 1 Interdisciplinary Program in Bioengineering, Seoul National University, Seoul 110-799, Korea; shongdr@gmail.com; 2 Department of Biomedical Engineering, Seoul National University College of Medicine, Seoul 110-799, Korea; 3 Institute of Medical & Biological Engineering, Medical Research Center, Seoul National University, Seoul 110-799, Korea

**Keywords:** unconstrained, ubiquitous, photoplethysmography, foot PPG, sole PPG

## Abstract

Photoplethysmography (PPG) of the foot sole could provide additional health-related information compared with traditional PPG of the finger or wrist. Previously, foot PPG required the procedural binding of a light-emitting diode (LED)-photodetector (PD) pair. We achieved PPG of the foot sole without binding any sensors to the foot while the participant stood in a natural standing position on the testing device. Foot PPG was performed using multiple LED-PD pairs to overcome motion artefacts caused by stabilization. We identified regions of the sole suitable for reliable sensor positioning with optimal LED-PD pairs on the basis of the estimated heart rate (HR) and signal quality index derived by dynamic time warping (wSQI). The first experiment included four participants with direct skin-to-sensor contact, and the results showed a mean HR estimation error of 0.01 beats/min and a wSQI of 0.909. The extended experiment with 53 participants, which involved including a gap between the skin and sensors to consider real-life applications, yielded a mean HR estimation error of 0.638 beats/min and a wSQI of 0.751. Based on the selection ratio of optimal LED-PD pairs, the best region of the sole for PPG was the midfoot, except the medial longitudinal arch. In conclusion, we confirmed that foot PPG using multiple LED-PD pairs is appropriate for HR evaluation and further applications.

## 1. Introduction

As a human stands naturally on a surface, it is possible to obtain information about the heart, blood vessels, and foot tissue without the person wearing any devices: this is the aim of unobtrusive photoplethysmography (PPG) of the sole of the foot. Foot photoplethysmograms include physiological information from the heart towards the lower extremities because PPG detects light variations originating from changes in the blood volume being transmitted from the left ventricle. Previous studies have reported various medical applications of foot PPG, including the monitoring of the vascular status, the prevention of diabetic foot ulcers [1,2], and the prediction of sympathetically mediated pain in the lower limbs to evaluate the success of pain-relieving treatments [3]. Foot PPG has another common medical application: detecting of peaks or valleys in cardiac cycles to identify arrhythmias (i.e., abnormal heart beats) and prevent sudden death due to a heart attack. The parameters of increased blood volume in limbs, including the pulse arrival time (PAT) and pulse transit time (PTT) in reference to the electrical or mechanical onset of a systolic event, have been investigated to estimate blood pressure without the pain and discomfort caused by occluding cuffs [4]. Additionally, compared with conventional finger PPG, foot PPG has been reported to be more accurate for estimating blood pressure and could provide robust measurements during tasks that involve smooth muscle contractions in the arm [5]. Despite its usefulness in healthcare, foot PPG is used much less than finger PPG. This difference is due to burdensome fixation of the foot sensor, reduced accessibility of the sensing modality, and its difficult application during daily activities. Stable light collection is conventionally maintained by attaching sensors to the foot with sticky materials or a binding strap. In previously published studies of foot PPG, most form factors required additional fixing procedures. For example, a sticky cover attachment was required for a pulse oximeter probe (N-25 Oxisensor II, NelcorPuritan Bennett Inc., Pleasanton, CA, USA) to analyse pulse transit time to the foot [6] and an early indicator of sympathectomy after epidural anesthesia in PPG [7]. A probe (probe type 75333-5, Artema, Albertslund, Denmark) required fixation with a velcro wrap-around cuff to inspect the variability of signals between measurement sites (i.e., toes, thumbs, and ears) [8], age-related changes in the characteristics of waveforms [9], lower limb peripheral arterial occlusive disease [10], and cardiovascular disease through foot PPG [11]. Therefore, measurements cannot be conducted under daily conditions, and analyses or clinical assessments cannot be performed based on accumulated data because of the low accessibility of this technique.

To enhance the opportunity for obtaining health-related information from the foot, we researched a method to eliminate the requirements of intentional measurement procedures: performing PPG at the sole of the foot under the weight of the body while standing in a natural posture. Humans adopt the standing state frequently during daily activities such as thinking, focusing on a task, and weighing oneself on a scale. A previous study of standing postures with a pressure sensor under the sole (i.e., ballistocardiography, BCG) revealed that an obstacle to unobtrusive foot PPG under the sole is stabilization activity during quiet standing [12]. The irregular movements to resist gravity obscure the sensitive physiological signals from the foot. One reason for such stabilizing movements is that the human mass, which is essentially an inverted pendulum, cannot be fully supported by two narrow feet and ankles. Thus, motion artefacts are created by the response of the triceps surae muscles to maintain balance by predictively controlling the proximal offset of the spring-like element, which is too compliant to achieve stability [13]. Another reason for these movements is the physiological activity (e.g., of the lungs and diaphragm) required for inhalation and exhalation. Although one study investigated foot PPG under the sole without compulsory contacts using mat-type sensors, the research conditions were limited to a participant in the sitting position for estimating blood pressure [14]; this study also obtained a signal with a standing posture, but its quality was notably worse than that obtained with sitting. Additionally, direct contact with protruding sensors caused discomfort and limited applications involving long-term measurements (e.g., through an insole or floor).

Therefore, we conducted this intensive study with various sensor options, including embedded designs, for real-life applications. Through the placement of multiple light-emitting diode (LED)-photodetector (PD) pairs, we successfully conducted standing foot PPG. Various comparisons of foot PPG signal quality were performed considering the locations of sensor contact with the sole. Optimizing the placement of the LED-PD pairs compensated for the reduced signal intensity and contact instability caused by physical gaps between the skin and the embedded sensors.

The remaining sections of the present paper are organized as follows: in Section 2, we describe the experimental methods used for standing foot PPG. We conducted two experiments, one with direct skin-to-sensor contact and one with gaps between skin and sensors. In Section 3, we describe our findings and discuss the foot PPG experiments. Section 4 concludes the present study.

## 2. Materials and Methods

In all, 57 healthy volunteers (34 men and 23 women) ranging from 22 to 60 years of age (mean (SD): 29.5 (5.9) years of age) participated in the PPG study. None of the participants had psychological or neurological disorders. All subjects provided their informed consent for inclusion before they participated in the study. The study was conducted in accordance with the Declaration of Helsinki, and the protocol was approved by the Institutional Review Board of the Seoul National University Hospital (IRB No. C-1703-045-837).

An MP150 data acquisition system (BIOPAC Systems, Inc., Goleta, CA, USA) simultaneously recorded a finger photoplethysmogram as a reference and the foot photoplethysmogram at a sampling rate of 500 Hz. As a reference, finger PPG was performed using a qualified PPG module (PPG100C, BIOPAC) with a paired (TSD200, BIOPAC) infrared (IR) LED and PD. A Velcro strap was used to fix the LED-PD pair to the middle fingertip of each participant’s right hand. The foot photoplethysmogram was recorded using a universal interface module (UIM100C, BIOPAC) and LED-PD pairs on a printed circuit board. 

### 2.1. Foot PPG Measurements

To obtain optimal foot PPG measurements, the following procedures were applied. First, the light wavelength of the source was matched to the sensitive spectral range of the detector to reduce the effect of ambient light noise. Second, multiple source-detector separation (SDS) and probe locations were prepared. Finally, the embedded design was applied to eliminate potential inconveniences caused by the sensors.

LEDs and PDs with matched light wavelengths in the near-IR range were selected to maximize light extinction by the blood in the sole of the foot and to minimize the light noise during the experiments. For foot PPG, the light source was an 890-nm LED (VSMF3710, Vishay Semiconductors, Mansfield, WA, USA) with a typical intensity of 10 mW/sr (1.4 V) when the forward current was 100 mA and the pulse time was 20 ms. We chose this LED because it has nearly zero radiant power in the visible light range (i.e., λ < 800 nm). A voltage regulator (lm1117T-adj, Texas Instruments, Dallas, TX, USA) drove the direct current of the LED.

Around the LED, eight PDs (TSL260R, AMS, Canberra, Australia) detected and transformed the light signals into amplified voltage signals. The TSL260R shows high sensitivity only from 880 to 960 nm with an integrated visible light cut-off filter. Through-hole plastic packaging of the PD with silicon moulding on the electro-conductive surfaces was used to prevent signal contamination by direct skin-to-sensor contact.

**Figure 1:**
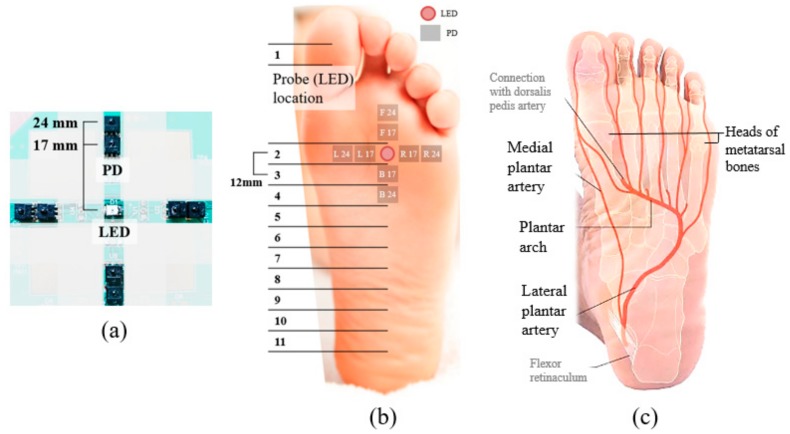
Foot PPG with direct skin contact based on the anatomical structure. (**a**) Probe for foot PPG. (**b**) Probe locations and PD placement. (**c**) Anatomical structure of the sole of the foot.

We located two PDs along four directions (i.e., left, right, forward, and backward) with an LED in the centre (Figure 1a). These four directions represent the medial, lateral, distal, and proximal directions, respectively, on the sole of the right foot. In each direction, the two PDs were 17 mm and 24 mm from the LED. We excluded shorter distances (e.g., 10 mm) because the intensity of the LED light exceeded the acceptable range of the PD at those distances.

The foot PPG probe (a single LED and eight PDs) was installed for the following two experiments. The two experiments mainly differed by the presence of a 0.5-mm gap between the skin and the sensor. For foot PPG, the participants stood with their feet shoulder-width apart, their right foot on the foot PPG board, and their arms hanging naturally at the sides. They were instructed to stand upright in a resting state (i.e., with relaxed body parts).

#### 2.1.1. Experiment 1: Foot PPG with Direct Skin-Sensor Contact

The goal of the first experiment was to confirm the feasibility of standing foot PPG. The prominent surface of the probe directly contacted the skin of the participant’s sole. We performed foot PPG with the probe at 11 locations (12 mm apart, Figure 1b) on the right foot of each of four healthy volunteers. The 11 horizontal locations were selected on the basis of the bone structure (Figure 1c) of the foot for reproducibility. We set the horizontal lines of the probe parallel to the line formed by the tibial sesamoid of the first metatarsal bone and the fifth metatarsal head. The participants were asked to stand still on the PPG board for 90 s.

#### 2.1.2. Experiment 2: Foot PPG with a Gap between the Skin and Embedded Sensors

**Figure 2:**
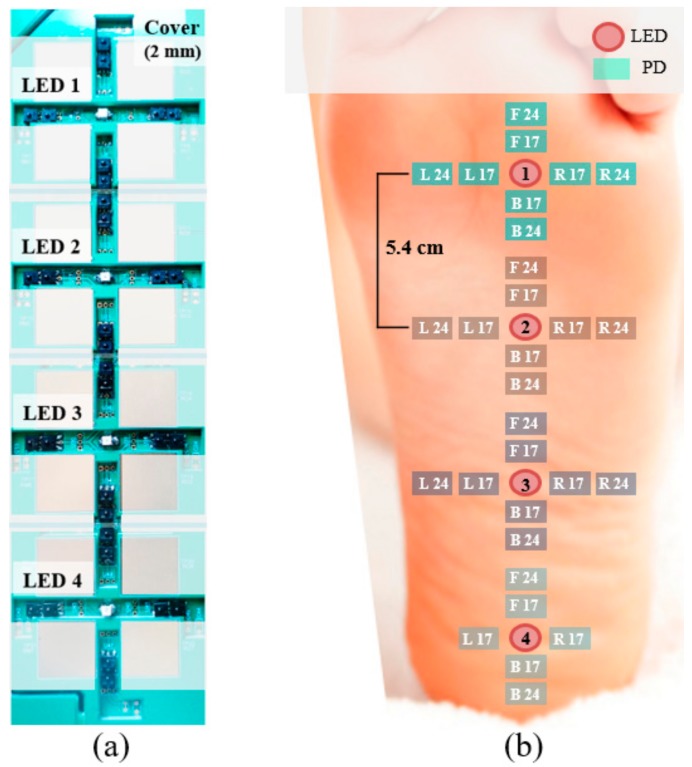
Foot PPG with a gap between the skin and embedded sensors. (**a**) Probes with a cover to form a gap between the skin and sensors. (**b**) Positions of four LEDs and 30 PDs.

A non-conductive cover with a thickness of 2 mm was used to create a 0.5-mm gap between the skin and sensors (Figure 2a). We tested four probe locations to compare the differences in signal quality as a function of the position of contact with the foot. The first probe (LED 1) was placed at location 2 from experiment 1. LED 2, LED 3, and LED 4 were positioned 54, 108 and 162 mm away from LED 1 along the midline of the right foot. The eight PDs around each LED were positioned as in experiment 1. A 32-channel data acquisition system (NI USB-6218, National Instruments, Austin, TX, USA) simultaneously measured signals from 30 LED-PD pairs (except L24 and R24 of the fourth probe, Figure 2b) and shared pulses with the BIOPAC system for synchronization with the reference PPG. The total measurement time for each of the 53 healthy participants (30 men and 23 women) in standing posture was 120 s. The length of the sole (i.e., from the heel end to the tibial sesamoid) of each participant was measured after the task.

### 2.2. PPG Valley Detection

**Figure 3:**
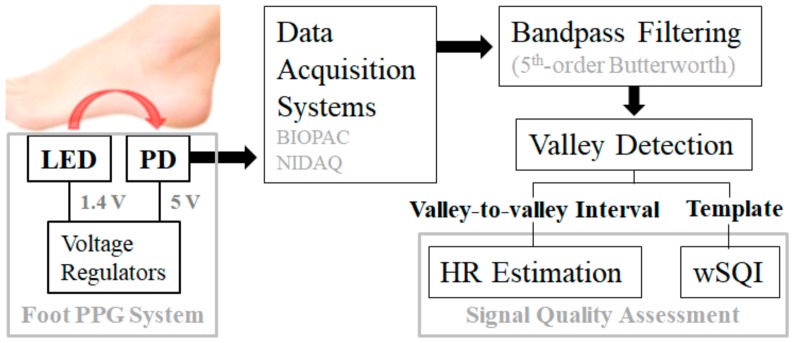
Process for signal quality assessments of foot PPG.

Systolic contraction of the left ventricle causes a phasic increase in the ejected blood volume and an increase in light absorption. During the diastolic phase, light absorption in peripheral limbs decreases with decreasing blood volume. At the end of diastole and the beginning of systole, the PPG waveform shows a so-called ‘valley’ or ‘foot point’, indicating the least light absorption. As shown in Figure 3, we detected valleys in the digitalized and filtered PPG waveform using a modified *wabp* algorithm [15] for a 50% overlapping 6-s sliding window. Using the valley-to-valley intervals in the window, we estimated the heart rate (HR) and calculated the index of similarity to the reference HR. Additionally, we derived signal quality indices using the cardiac cycles between two consecutive valleys of the measured PPG waveform. Analysis of the signal quality metrics allowed selection of an optimal LED-PD pair.

To enhance the robustness of the valley detection and assessments of signal quality, we applied a prior filtering process to extract signals related to the heartbeat. We cascaded low-pass and high-pass filters with approximation functions of Butterworth design to minimize amplitude distortions. We took this approach because the pass band (i.e., from 0 Hz to the cut-off frequency at −3 dB) of the Butterworth filter is mathematically designed to have a maximally flat frequency response. If we define *j* as equal to the square root of −1 and omega (*ω*) represents an angular frequency, the gain, *G*(*ω*), of an *n*-order Butterworth low-pass filter can be represented as:

(1) $$G^{2}(\omega )={\left|\frac{V_{out}(j\omega )}{V_{in}(j\omega )}\right|}^{2}={\left|H(j\omega )\right|}^{2}=\frac{G_{0}^{2}}{1+{\left(\frac{j\omega }{j\omega_{c}}\right)}^{2n}},$$

where *G*_0_ is the gain at 0 Hz (direct-current), *n* denotes the filter order, and *ω_c_* represents the cut-off angular frequency.

The derivative of *G*(*ω*) with respect to angular frequency can be calculated as:

(2) $$\frac{dG}{d\omega }=-nG^{3}\omega^{2n-1}\;$$

Because the gain is always positive, Equation (2) shows the theoretical absence of distortion by filtering in the pass band. On the other hand, the output of the high-pass filter (used to remove baseline drift) is mathematically equal to the intermediate result of subtracting a low-pass filter from an all-pass filter. Using the Butterworth design, bandpass filtering (i.e., a fifth-order filter with a pass band of 0.4–2.9 Hz) was performed. The first 30 s of data were excluded from all filtered PPG waveforms to eliminate transitional states.

### 2.3. Similarity of Estimated HR to Reference HR

One important application of PPG is HR estimation using intervals between consecutive valleys (beats). Each time, the index of a detected valley, $v_{n}$, can be stored in a matrix *V* for each channel (*ch*, LED-PD pair):

(3) $$V_{ch}=[v_{1},\ldots ,v_{n}].\;$$

The HR can be calculated using valley-to-valley intervals (*VVIs*) with differences between adjacent valleys as follows:

(4) $$VVI_{ch}=[vvi_{1},\ldots ,vvi_{n-1}]=[v_{2}-v_{1},\ldots ,v_{n}-v_{n-1}]\;(s),$$

$$HR_{ch}=60/VVI_{ch}\left(\mathrm{beats}/\min\right).$$

The ground truth HR values were calculated using the fixed channel of the reference finger PPG (*HR_ref_*). We defined the HR error (*HR_err_*) as the absolute difference between the reference HR and the HR estimated by foot PPG (*HR_foot_*):

(5) $$HR_{err}=\left|HR_{ref}-HR_{foot}\right|\;\left(\mathrm{beats}/\min\right).$$

If we define the similarity of the HR as the ratio of *HR_ref_* to *HR_est_*, the index cannot be normalized because either of the HR values could be higher than the other. Therefore, we defined the HR similarity index as the ratio of *HR_ref_* to the sum of the HR error and *HR_ref_*. This index was normalized to a maximum of 100% regardless of whether *HR_ref_* or *HR_foot_* was higher as follows:

(6) $$SI_{HR}=\frac{HR_{ref}}{HR_{ref}+HR_{err}}\cdot 100\;(\%).$$

Furthermore, we created linear regression and Bland-Altman plots [16] to visually represent the comparisons in experiment 2. These plots commonly showed the agreement between the new measurement technique and the reference method. In particular, the Bland-Altman plot was illustrated by the Cartesian coordinates (*BA*(*x,y*)) of derived values (i.e., the arithmetical mean and subtraction) using *HR_ref_* and *HR_foot_*:

(7) $$BA(x,y)=(\frac{HR_{ref}+HR_{foot}}{2},HR_{ref}-HR_{foot}).$$

The y-axis values represent the HR errors obtained by foot PPG. Horizontal dotted lines showing the 95% limits of agreement in the comparisons (the average difference ± 1.96 standard deviation [SD] of the difference) show the distribution of the HRs in the two simultaneous PPG measurements.

### 2.4. Quantification of PPG Quality

To obtain reliable health-related information, the reliability of the signal must be ensured by assessing the quality of the measured photoplethysmogram. We applied correlation-based signal quality indices (SQIs) for the assessment of signal quality [17]. The SQIs were commonly derived from the cross-correlation of every heartbeat signal using a template. We defined the template as the averaged waveform of the reference photoplethysmogram between two normal valleys in a 6-s window. The two time series datasets, the beat template (*T*) of reference PPG and the signal of foot PPG (*F*), were represented by:

(8) $$T=[t_{1},t_{2},\ldots ,t_{i},\ldots ,t_{N}]$$

(9) $$F=[f_{1},f_{2},\ldots ,f_{j},\ldots ,f_{M}].$$

Three SQIs were defined: oSQI, the cross-correlation coefficient based on direct matching of the original signals to the template; rSQI, the cross-correlation coefficient derived after linear resampling to compress or stretch the foot PPG signal such that the length of a beat (*M*) would be equal to the length of the template (*N*); and wSQI, the cross-correlation coefficient estimated by dynamic time warping (DTW). The aim of DTW was to identify optimal alignments that yield the minimum cumulative distance between corresponding samples from (0,0) to (*N*,*M*) in the two time series. The distance *d*(*t_i_*, *f_j_*) between the two sample points *t_i_* and *f_j_* was the (*i*th, *j*th) element of an *N* × *M* distance matrix (D).

Using a piecewise linear approximation algorithm [18], the template and one beat signal of a foot PPG were transformed to distance sequences. The absolute difference between the slopes of each line represented the distance between each ‘line pair (*d*(*t_i_*, *f_j_*))’. If we define *l*(*t_i_*) and *l*(*f_j_*) as the time durations of lines *t_i_* and *f_j_*, the cumulative distance, *C_i,j_* (up to lines *i* and *j*), can be defined as:

(10) $$c_{i,j}=\min\;\{\begin{array}{c} {}_{c_{i-1,j}+d(t_{i},f_{j})l(t_{i})}\\ {}_{c_{i-1,j-1}+d(t_{i},f_{j})\{l(t_{i})+l(f_{j})\}}\\ {}_{c_{i,j-1}+d(t_{i},f_{j})l(f_{j})} \end{array}$$

DTW can compensate for the structural difference in the pulse transit route from the aorta and the cardiovascular state with a minimized *C_i,j_*. Therefore, DTW might be suitable for estimating correlations between the foot and reference finger photoplethysmograms [17,19]. In the following results, we used wSQI as the representative quality index.

### 2.5. Specification of a Foot PPG System and Processing Time

The sizes of the PPG probes were 4.75 × 4.75 cm^2^ (one LED and eight PDs) and 21 × 4.75 cm^2^ (four LEDs and 30 PDs). The voltage regulators were implemented in an 8 × 8-cm^2^ circuit board. To analyse the practical power consumptions of the present system, we conducted battery life tests with two 11.1-V lithium polymer batteries with different capacities, 0.65 Ah (52 × 35 × 12 mm^3^) and 4 Ah (140 × 55 × 16 mm^3^). The operating times of a foot PPG probe (one LED and eight detectors) were 17 hours with a 0.65-Ah battery and approximately 4 days with a 4-Ah battery.

The process for deriving signal quality metrics was performed using MATLAB R2017b technical computing software (MathWorks, Natick, MA, USA) running with an Intel^®^ Core™ i7-7700 3.6-GHz processor with 24 GB RAM. The process time for valley detection, HR estimation, and wSQI calculation of a channel (LED-PD pair) was 0.5 s. The total process time for the 30-channel datasets was 14.1 s. Bandpass filtering in the procedures required 0.2 s. The replacement of software filtering by hardware filtering would reduce the preprocessing time.

## 3. Results and Discussion

### 3.1. Experiment 1

**Figure 4:**
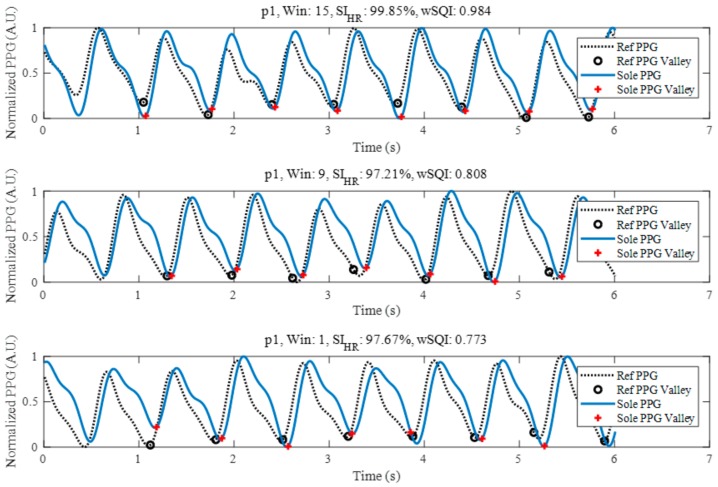
*SI_HR_* and wSQI as a function of the time window of foot PPG of participant 1 (p1) with probe location 2, PD F17 and direct skin contact.

We determined the feasibility of performing foot PPG in the standing position, as shown in Figure 4. The three examples plotted are in descending order of the wSQI derived from PD F17 at probe location 2 for participant 1 during direct sole contact. At the top of Figure 4, the data show a wSQI greater than 0.9. Most foot photoplethysmograms showed similar morphologies in relation to the reference PPG. Even for morphologies such as that shown in the middle of Figure 4, which show larger variations in the amplitude of the foot PPG signal than in that of the reference PPG signal, the wSQI was approximately 0.8. The foot PPG signal in the bottom of the figure, with a high *SI_HR_* of 97.67% and a low wSQI of 0.773, indicates the high accuracy of the valley detection. It also shows that the correlation-based SQIs do not always vary dependably with each other because the correlation coefficient is sensitive to not just the valley points but also the overall morphology of the PPG signal based on amplitude variations.

**Figure 5:**
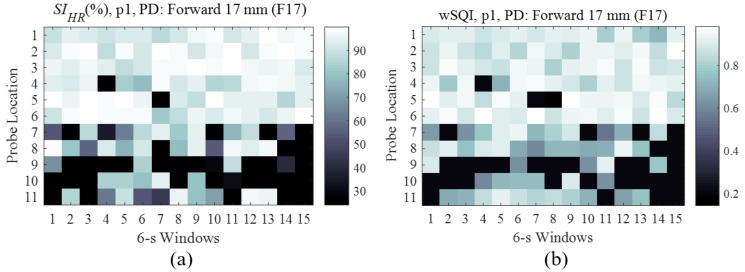
Indices according to probe locations in p1 and a 6-s windows: (**a**) *SI_HR_* and (**b**) wSQI.

For each PD, the *SI_HR_* (Figure 5a) and wSQI (Figure 5b) of each PD can be illustrated by a heatmap according to the probe location (y-axis) and time window (x-axis). The brightness of each square of coordinates indicates the quality of foot PPG (*SI_HR_* and wSQI) using a PD (F17) as a function of the probe location and time window. In these figures, we compared the contact stability of the probe locations with time windows varying from 1 to 15 s. We confirmed that the stability of the contacts varied among the time windows. If the brightness at a probe location was generally stable, the location was applicable for foot PPG measurements. For F17 and participant 1 (p1), the stable contact at probe location 2 yielded the lowest HR error with the highest mean *SI_HR_* (99.99%) and a wSQI of 0.897 for all time windows.

After averaging the indices for all time windows, we inspected the oSQI, rSQI and wSQI of the PD F17 obtained with p1, as shown in Table 1. The three SQIs varied in a similar manner depending on the probe location.

Overall, the wSQI was highest among the SQIs, as expected, due to the inherent difference in the blood transit routes between the hands and feet. By examining the SQIs, the highest mean wSQI of 0.927 indicated that probe location 6 was ideal for measuring foot PPG in p1 with F17.

Using procedures similar to those used to identify the best HR estimation, we found the optimal LED-PD pair for each individual participant (Table 2). The mean absolute error of 0.013 beats/min (BPM) demonstrated the usability of foot PPG for estimating HR. The mean wSQI derived from those LED-PD pairs was 0.909. The co-occurrence of the highest mean SIHR (99.9%) was observed in the individually averaged results for optimal combinations. The optimal probe locations varied, and locations 2, 7, 3, and 10 were optimal for different participants. According to these results, multiple LED-PD pairs are required to obtain reliable health-related information by foot PPG.

Considering the demand to choose only one or two optimal LED-PD pair positions, we inspected the mean *SI_HR_* for all participants (Table 3). The average *SI_HR_* for all probe locations was 87.9% for PDs at 17 mm and 92.9% for PDs at 24 mm, indicating that the 24-mm SDS was generally better than the 17-mm SDS for foot PPG with direct skin-to-sensor contact. The combination of probe location 3 and PD R24 yielded the highest mean *SI_HR_* (99.1%) and the lowest mean HR difference (0.89 BPM) for all participants. As a second optimal combination, PD F24 at the same probe location showed a mean *SI_HR_* of 98.4%. To select the most effective probe location for PDs in all four directions, the rankings of the averaged *SI_HR_* values for all PD combinations were inspected. The top five values were derived from probe locations 3, 8, 7, 6, and 2.

**Figure 6:**
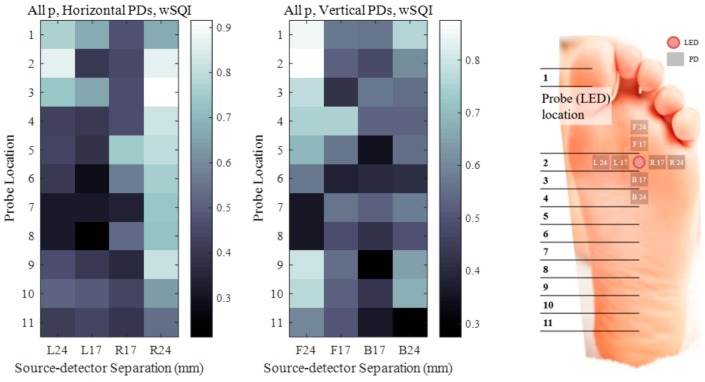
Foot PPG wSQI as a function of probe location and PD distance.

Figure 6 illustrates heatmaps of the wSQI averaged over all participants for horizontal PDs and vertical PDs. The y-axis represents the probe locations, and the x-axis shows each PD position from the LED on the midline of a right foot. This figure provides insight regarding the PPG quality obtained with each sensor position on the foot structure. Brighter and darker colours for the PD positions on a foot indicate higher and lower wSQI values, respectively. For example, the wSQI averaged over all time windows for PDs R24 and F24 was greater than 0.7 at location 3 (0.735) and location 9 (0.732), as shown in Figure 6.

### 3.2. Experiment 2

#### 3.2.1. Performance Based on Optimal LED-PD Pair Selections

We determined the effect of a gap between the foot and sensors by comparing the results of experiment 2 with those of experiment 1, which involved direct skin-to-sensor contact. The optimal channels for each time window were selected to analyse reliable physiological information. Table 4 presents the mean (SD) of the number of times LED-PD pairs were selected (selected counts) to show the minimum mean HR error among 53 participants for 40 time windows.

Using these optimal combinations for each window resulted in a total mean HR error of 0.638 BPM and a mean wSQI of 0.751. These results demonstrate the comfort of this device for foot PPG with a gap between the skin and sensors. The most frequently selected light source was LED 3. The mean (SD) of the dominantly selected counts was 7.4 (8.3) for R24 and 5.9 (7.9) for B24 with LED 3 among the 30 PDs. In general, the 24-mm SDS and right PDs for both LEDs were optimal for foot PPG. No left PDs from LED 2 were ranked in the top 12 due to the medial longitudinal arch of the right foot axis, as indicated in experiment 1.

**Figure 7:**
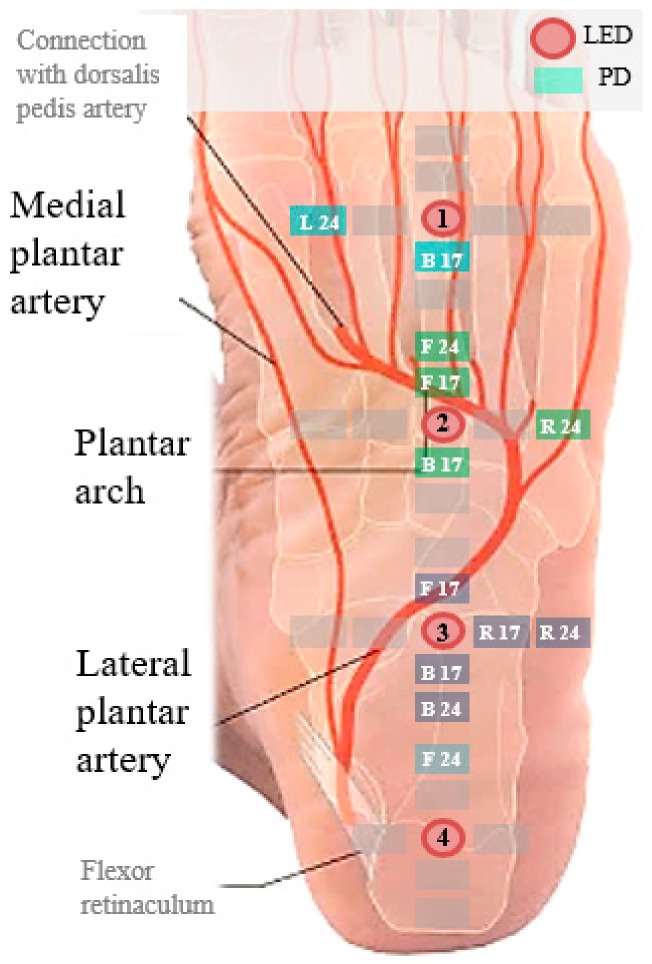
Top 12 LED-PD combinations superimposed on anatomical structure of the sole.

Figure 7 shows the top 12 combinations of these PD locations superimposed on the anatomical structure of the sole. The highest signal quality of the sensors was obtained near the lateral plantar artery and plantar arch between the anterior longitudinal sulcus and the heel. One reason for this finding is that the large blood volume changes in these blood vessels enhances the signal-to-noise ratio. The reason for the superior signal quality of the right PDs is the stability of the contacts. This contact stability is related to the bone structure of foot. The skin beneath the bones (e.g., calcaneus, cuboid, and fifth metatarsal base) from the heel to the fifth metatarsal head maintains steady contact with the ground. This finding is related to the results from a published study that attributed the stiffness during quiet standing to the foot, Achilles tendon and aponeurosis rather than the activated calf muscle. The authors measured the intrinsic mechanical stiffness of the ankle to come to this conclusion [13]. Muscular control is required to maintain balance during standing. The anatomical structure proximal to the heel bone provides more stability to the heel bone than to the affected toes.

We counted all optimal cases for each LED to obtain the selection ratio of the LEDs and a wider view of the sole regions (Table 5). Similar to the analysis of all PDs, LED 3, with a ratio of 49.9%, was dominantly selected. It was dominant because in addition to R24 and B24, F17 and B17 for LED 3 were ranked in the top 10. Thus, a probe placed with its centre 104 mm from the metatarsal heads is generally useful for PPG with a 17-mm SDS. Additionally, we calculated the mean (SD) length of the right soles of participants who showed an LED selection ratio exceeding 25% among the four LEDs. In all, we selected 9.3% of the combinations with LED 4 for soles, with the highest mean length of 181 mm. The sole length particularly affected contact with probe 4. The centre of this probe was 162 mm from the metatarsal heads. We noted that F24 with LED 4 was ranked seventh. The distal area of heel near the lateral plantar artery is thus suitable for PPG because the sole lengths of only 13 participants were greater than 184 mm. PD B24 of probe 3 ranked second. It contacted the distal area of the heel in participants with short soles and the proximal area of the midfoot in participants with long soles. In contrast, the ball of the foot was less suitable for PPG because the fat pads underneath the metatarsal heads for protecting the foot reduced the distance between the blood vessels and sensors.

**Figure 8:**
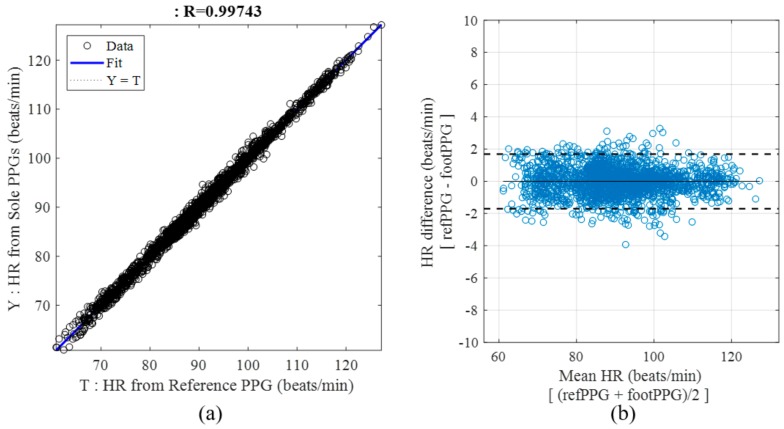
Scatter plots of HRs estimated by foot PPG and reference HRs with 40 time windows for 53 participants. (**a**) Linear regression plot. (**b**) Bland–Altman plot.

The HRs for 40 time windows in 53 participants (i.e., 2120 samples) with optimal LED-PD pairs are shown as scatter plots. Most HRs were distributed around the ideal line that represents equality between the HR estimated by foot PPG (Y) and the target HR of the reference PPG (T) (Figure 8a). The mean (SD) HR of the whole dataset was 90.9 (11.5) BPM. The cross-correlation coefficient derived from the reference and estimated HRs was greater than 0.994. These findings indicate that the estimated HRs were an accurate reflection of the reference HRs, according to the variations in each participant’s HR. The Bland-Altman plot (Figure 8b) shows that considerable data are distributed within the dotted lines with a difference of approximately 2 BPM. Some unusual HR errors were observed due to unstable contact; however, the mean HR errors for all participants obtained using the optimal sensor combinations shown in Table 4 were less than 0.9 BPM.

#### 3.2.2. Effect of Standing Time, Age and Sex on Performance

Considering the relationship between leg fatigue and contact stability with long standing durations, we analysed the coverages in the range of wSQIs over different standing periods (Table 6). We conducted two-tailed paired *t*-tests with the null hypothesis that the mean wSQI coverage in the first half (1~20 windows) and the second half (21~40 windows) of the experiment were not different. The coverage of each wSQI range was not significantly different (mean *p*-value: 0.59) among different elapsed times. At least 126 s of task time did not significantly affect the foot PPG signal quality despite a 1.7% decrease in the coverage of the range of wSQI over 0.8.

We also applied the Wilcoxon rank-sum test (or Mann-Whitney U-test), which is designed to analyse the distributions of two groups of different sizes. We derived the *p*-values from statistical tests with a null hypothesis that the means of the two groups were not different. The two types of groupings were as follows.

First, we grouped the participants to balance them based on age (i.e., 27 years of age). The left column in Table 7 shows the average (SD) of the signal quality metrics of the foot PPG signal for each group according to age. The mean (SD) HR was 90.21 (10.16) BPM for participants younger than or equal to 27 years and 91.6 (12.42) BPM for participants older than 27 years. Non-significant values (*p* > 0.05) for *HR_err_* (*p* = 0.66), *SI_HR_* (*p* = 0.65), and wSQI (*p* = 0.51) were obtained by the Wilcoxon rank-sum test. 

Second, we analysed the PPG data grouped by sex, as shown in the right column in Table 7. The mean (SD) HR was 92.05 (12.16) for men and 89.44 (10.1) for women. The Wilcoxon rank-sum test gave *p*-values greater than 0.05, indicating that the *HR_err_* (*p* = 0.66), *SI_HR_* (*p* = 0.41), and wSQI (*p* = 0.54) were not significantly different between the sexes.

### 3.3. Comparison with Unobtrusive Physiological Measurement Studies

Previous studies analogous to this study have measured physiological signals without the fixation of sensors [20]. Remote and non-contact measurements (e.g., facial videos and radar) showed a mean HR error greater than 3 BPM [21,22]. For example, when gravity was used in a seat system without direct skin-to-sensor contact, the oSQI (i.e., cross-correlation coefficient at zero lag) was greater than 0.87 for all 6-s segments despite light attenuation through clothing [23].

Table 8 shows the HR-related signal quality metrics of previously published unobtrusive measurement studies. In particular, using gravity and the standing posture, BCG with an electronic weighing scale produced an error of ±21 ms (approximately 2 BPM) as the 95% confidence interval for the interbeat interval (IBI) in 17 participants after the removal of contaminated data from three participants in a dataset of 20 participants [24]. Plantar impedance plethysmography (IPG) in the standing posture resulted in an error of ±30.65 ms (approximately 3 BPM) as the 95% confidence interval for IBI in 10 participants [25]. With contacts through clothing on a chair system, capacitively coupled electrocardiography (ECG) (back), PPG (thigh), and BCG (hip) achieved mean HR errors of 0.034, 0.640, and 1.857 BPM, respectively [26]. The mean HR error of the present study (i.e., 0.638 BPM) is similar to the PPG result obtained from the thigh through clothing and is better than the BCG result measured in participants sitting and leaning back on a chair.

The scatter plots of the HRs with optimal LED-PD pairs informed us of the potential and limitations of foot PPG performed under the sole with a gap between the skin and sensors. The 95% confidence interval of 2 BPM indicates that these measurement values are better than or similar to the results of previously published unobtrusive measurement studies. Furthermore, this confidence interval demonstrates the excellence of foot PPG for monitoring HRs, considering the HR error range of commercial wrist-worn PPG-based systems [27]. The wrist-worn systems showed a mean absolute percentage error of 2.8–5.4% in estimating HRs during rest, even with sensor band binding. These bands should be loosely tied to avoid putting long-term pressure on the wrist under real-life conditions. Although the reasonable signal quality of foot PPG is similar to that of commercial bands, the limitation of foot PPG was the discomfort caused by direct contact with prominent sensors. This challenge might limit the application scope of this technique. In particular, form factors with long-term contacts (e.g., insole or floor) in the standing state might cause pain in the sole of the foot. We can overcome this discomfort by using a cover to make the surface flat. However, the physical gap caused by the cover increased the mean HR error compared with that obtained with direct contact. Errors related to fiducial points can cause critical failures in analysing health-related information (e.g., blood pressure estimation based on the PAT and PTT). Therefore, a trade-off exists between comfort, which allows long-term use, and measurement accuracy. The present study confirms that the use of multiple sensors increases the robustness of the measurements when a physical gap is present and the stability of contacts with each foot structure varies. Another experimental finding was that different anatomical regions of the sole showed various light amplitudes depending on the sensor positions. The signal quality can be improved if we adapt the optimal light intensity for each probe location in further research. This objective can be achieved with automatic lighting controls based on prior information such as signal quality metrics.

Additionally, even the morphology of the PPG waveform must be clear to allow data extraction from a body area sensor network using a biometrics approach [28]. In these applications, which require high-quality waveform morphologies, direct skin-to-sensor contact is recommended for obtaining reliable information. With templates derived from accumulated clean data, the SQI can allow the extraction of reliable data even when the reference PPG cannot be measured.

Continuous or frequent measurements of zero-effort foot PPG might facilitate an accurate and rapid diagnosis of health problems. Among abnormal health statuses, chronic diseases, including heart problems and serious diabetes, require regular diagnosis. The present results are limited to participants without chronic disease because the study focused on confirming the potential of foot PPG. Assessments of chronic diseases using foot PPG would yield more practical results in future studies.

## 4. Conclusions

Utilizing multiple LED-PD sensors, we demonstrate the possibility of standing foot PPG for u-healthcare applications without the requirement of bindings. We analysed various areas for the measurements based on various signal quality metrics, and the midfoot from the metatarsal heads to the distal area of heel was identified as the optimal location for foot PPG. The optimal selection of multiple sensor channels enhanced obtaining reliable health-related information. Considering all the experimental results, the sensing structure (direct or indirect skin-to-sensor contact) and data preprocessing procedures must be determined according to the signal reliability requirements of each application. The foot PPG signal quality would be improved by individually designing the sensor probe for each participant by considering his/her various foot structures, obtained by physical anthropometry (i.e., individual body measurements). Through these procedures, foot PPG could accumulate cardiac and vascular information unobtrusively on a daily basis. Plentiful PPG data from zero-effort measurements could enable the avoidance of chronic diseases and the early detection of acute episodes during daily life.

## Figures and Tables

**Table 1:** Comparison of the mean SQI values obtained for p1 with F17 for all time windows.

Probe Location	oSQI	rSQI	wSQI
1	0.743	0.745	0.882
2	0.763	0.766	0.897
3	0.831	0.835	0.906
4	0.729	0.726	0.846
5	0.708	0.712	0.827
6	0.915	0.918	**0.927**
7	0.436	0.438	0.613
8	0.468	0.464	0.704
9	0.269	0.277	0.402
10	0.260	0.258	0.408
11	0.272	0.276	0.628

**Table 2:** LED-PD pairs yielding minimal HR errors in the direct-contact foot PPG experiment.

Participant	*HR_err_* [*BPM*]	wSQI	Probe Location	PD Position
p1	0.002	0.897	2	F17
p2	0.002	0.881	7	F17
p3	0.011	0.898	3	F24
p4	0.038	0.959	10	B24
Average	0.013	0.909		

**Table 3:** Mean HR similarity index [%] for all participants and each LED-PD pair.

Probe Location	Horizontal PDs				Vertical PDs			
	L24	L17	R17	R24	F24	F17	B17	B24
1	92.5	87.5	88.0	92.0	92.5	87.4	91.4	93.8
2	97.9	86.3	85.7	97.6	97.5	85.5	89.4	90.4
3	93.9	96.8	85.9	**99.1**	**98.4**	90.0	93.8	92.6
4	86.6	90.7	87.1	96.7	89.0	90.8	89.7	88.1
5	89.2	83.6	92.0	95.8	92.2	90.9	80.4	88.2
6	93.6	84.1	90.6	94.2	95.3	91.7	90.4	91.0
7	94.0	87.1	85.9	94.8	94.8	90.4	92.2	92.9
8	92.3	92.6	88.7	94.3	94.4	95.0	88.2	90.0
9	88.0	87.5	79.7	94.3	93.5	87.3	83.4	89.1
10	92.3	92.4	81.5	94.5	95.6	88.4	81.6	92.1
11	92.0	85.4	82.8	88.6	93.9	85.4	83.0	86.9

**Table 4:** *HR_err_*, *SI_HR_* and wSQI for all participants from the top 12 LED-PD pairs with a gap between the skin and sensors.

Selected Count Mean (SD)	LED	PD	*HR_err_* Mean (SD)	*SI_HR_* [%] Mean (SD)	wSQI Mean (SD)
7.4 (8.3)	3	R24	0.56 (0.40)	99.4 (0.4)	0.845 (0.065)
5.9 (7.9)	3	B24	0.58 (0.41)	99.3 (0.4)	0.883 (0.054)
3.8 (6.5)	2	R24	0.66 (0.45)	99.2 (0.6)	0.880 (0.068)
3.8 (6.7)	2	F24	0.81 (0.34)	99.1 (0.4)	0.859 (0.074)
2.8 (6.3)	3	F17	0.53 (0.43)	99.3 (0.5)	0.841 (0.076)
2.0 (3.2)	1	B17	0.77 (0.47)	99.1 (0.6)	0.856 (0.068)
2.0 (3.7)	4	F24	0.73 (0.44)	99.1 (0.5)	0.819 (0.075)
1.0 (2.4)	2	F17	0.86 (0.45)	99.0 (0.6)	0.818 (0.058)
1.0 (2.7)	2	B17	0.73 (0.52)	99.2 (0.5)	0.886 (0.050)
0.9 (1.7)	3	B17	0.81 (0.65)	99.0 (0.8)	0.825 (0.077)
0.8 (2.1)	1	L24	0.66 (0.48)	99.2 (0.6)	0.834 (0.079)
0.8 (1.6)	3	R17	0.75 (0.48)	99.2 (0.5)	0.833 (0.084)

**Table 5:** Selection ratio for each LED for sole lengths from the heel end to the metatarsal line.

LED Number	Selection Ratio (%)	Sole Length (mm) Mean (SD)	Cases of Selection Ratio > 25%
**1**	12.1	173 (10)	7
**2**	28.7	173 (11)	22
**3**	49.9	174 (12)	36
**4**	9.3	181 (11)	6

**Table 6:** Mean coverage [%] for wSQI ranges, depending on the standing duration. We determined the *p*-values by a *t*-test with the null hypothesis that the mean wSQI coverage was not different between windows 1~20 and windows 21~40.

Signal Quality Criteria	Standing Period		
	Windows 1~20	Windows 21~40	*p*-Value
**wSQI > 0.6**	99.0%	98.5%	0.29
**wSQI > 0.7**	94.5%	94.1%	0.47
**wSQI > 0.8**	75.8%	74.1%	0.22

**Table 7:** Wilcoxon rank-sum test results and mean (SD) signal quality metrics of different groups.

Signal Quality Metric	Age			Sex		
	≤27 (N = 26)	>27 (N = 27)	*p*-Value	Men (N = 30)	Women (N = 23)	*p*-Value
*HR_err_* (BPM)	0.66 (0.31)	0.61 (0.31)	0.66	0.63 (0.34)	0.65 (0.27)	0.66
*SI_HR_* (%)	99.2 (0.36)	99.3 (0.38)	0.65	99.3 (0.4)	99.2 (0.33)	0.41
wSQI	0.859 (0.04)	0.871 (0.03)	0.51	0.867 (0.04)	0.862 (0.03)	0.54

**Table 8:** Performance of unobtrusive measurements in previous studies.

Authors	Modality	Quality Metrics	Performance	Participants
González-Landaeta et al., 2008	BCG by weighing scale in the standing posture	Absolute 95% confidence interval of IBI	21 ms	17 (N.A.)
Diaz et al., 2010	IPG under the plantar region in the standing posture	Absolute 95% confidence interval of IBI	30.65 ms	10 (3 women)
Baek et al., 2012	CCECG (back) PPG through clothing (thigh) BCG while leaning back on a chair	Mean HR error	0.034 BPM (CCECG) 0.640 BPM (PPG) 1.857 BPM (BCG)	5 men

N.A.: Not available.
